# The Dark Triad and Professional Fighters: Destigmatizing Male Combat Athletes

**DOI:** 10.7759/cureus.33219

**Published:** 2023-01-01

**Authors:** Olivier Van Hauwermeiren, Jordan Kwamanakweenda, Julian Pino, Corey Peacock, Jaime L Tartar, Alexandru Cuc

**Affiliations:** 1 College of Psychology, Nova Southeastern University, Davie, USA; 2 College of Health Care Sciences, Nova Southeastern University, Davie, USA; 3 Psychology and Neuroscience, Nova Southeastern University, Davie, USA

**Keywords:** muay thai, personality, athletic performance, psychology, boxing

## Abstract

Personality characteristics have long been studied in relation to athletes and their performance. Of these, emotional self-regulation has been determined to be an important factor regarding outcomes for athletes. Various research has examined the Dark Triad (Machiavellianism, narcissism, and psychopathy) among other personality traits in athletes. However, little research has examined Dark Triad levels in combat sports professionals and their impact on their athletic performance. Since professional fighting athletes have been perceived as aggressive and impulsive, it is essential to examine the athletes’ level of Dark Triad traits. The present exploratory study investigates the association of Dark Triad personality traits in professional fighters versus college-aged male non-fighters. Secondly, it aims to evaluate the relationship between Dark Triad traits and winning percentages in professional fights. The study used a sample of professional male fighters (n = 36) and college-aged non-fighters (n = 29). Participants completed measures examining emotional self-regulation, trait anxiety, sensation seeking, and Dark Triad personality traits. The results of our study showed no significant differences in Machiavellianism, psychopathy, and narcissism between our samples of professional fighters and college-aged nonfighters. The study also showed no significant correlations between winning percentages for professional fighters and Machiavellianism, psychopathy, or narcissism.

## Introduction

Combat sports is an umbrella term for extreme striking or grappling sports such as wrestling, boxing, kickboxing, judo, and Muay Thai, amongst others. While some professional fighting disciplines, such as boxing, have been normalized within many cultures/countries, certain combat sports have been the target of recent scrutiny. Mixed martial arts (MMA) is a relatively new combat sport in which practitioners compete in contests to identify the best hand-to-hand combat techniques and the best combat sports practitioners. Conducted predominantly in so-called ‘cages’ (rings fenced in by metal net/mesh), the practice involves a wide range of striking techniques, including knees and elbows, and grappling techniques, such as chokes, takedowns, and submission holds. Without visual symbols of traditional safety measures, such as boxing gloves, headgear, or pads, along with a greater amount of visible blood due to superficial cuts, organizations engaged in early MMA events were stigmatized in various ways [1].

Personality traits have been studied extensively in various sports [2,3]. Among athletes, particular traits have been shown to predict various cognitive, affective, and behavioral outcomes [2]. The ‘Dark Triad’ refers to three distinct socially aversive personality traits of narcissism, Machiavellianism, and psychopathy as related to sports performance [4]. Despite their distinctive theoretical roots, the definitions of narcissism, Machiavellianism, and psychopathy are broad, and their distinction has become cloudy. In 2002, Paulhus and Williams [3] coined the term Dark Triad to encourage researchers to study the three traits in tandem: only then can their differences be distinguished. Prior research of the Dark Triad indicated several group differences; 1) gender: men scoring higher than women, 2) expertise: athletes with greater expertise scoring higher than those with less expertise and 3) type of sport: individual athletes scoring higher than team athletes across all factors on all three Dark Triad traits [4]. However, no research on the relationship between the Dark Triad and sports performance has included professional fighters. 

González-Hernandez et al. [5] researched the productive relationship between the Dark Triad and competitiveness. Additionally, they sought to identify differences in dark triad scores between professional and amateur athletes. Their study sampled 806 Spanish athletes that represented 11 different sports. Relevant to the current study, the results show that competitiveness is strongly related to the traits of the dark personality triad. The results suggested that dark personality traits are related not only to the individuality of the athletes but also to the self-perception of both their psychological response and competitiveness in their sporting environment [5].

Arthur et al. [6] investigated the role of athlete narcissism in moderating the relationship between coaches' transformational leader behaviors and athlete motivation. The results indicated that transformational leader behaviors that are less likely to provide individual self-enhancing opportunities and glorification have less impact on narcissists than those relatively low in narcissism. In addition, narcissism moderates the effect of fostering acceptance of group goals on effort, such that low narcissists were more positively affected by this behavior than high narcissists [6].

In essence, the results revealed that transformational leader behaviors that are less likely to provide opportunities for individual self-enhancement and glorification have less impact on narcissists than those relatively low in narcissism [6]. Furthermore, it demonstrated that narcissists would withhold effort when group goals are being sought [6]. Narcissists desire to be seen as outperforming expectations, and this perception of superiority is thought to be a key component in maintaining otherwise fragile self-esteem [7].

Despite their distinctive theoretical roots, narcissism, Machiavellianism, and psychopathy have overlapping characteristics that can blur the distinction between them. Paulhus and Williams coined the term Dark Triad to encourage researchers to study the three traits in tandem to clarify their distinctiveness [8]. 

The current exploratory study aimed to examine the Dark Triad personality traits among individual professional fighters and compare them to a non-fighting control group. We hypothesized significantly higher Machiavellianism, narcissism, or psychopathy scores between professional fighters and non-fighters. Furthermore, no current research investigates the relationship between Dark Triad variables and winning percentages; therefore, we incorporated an analysis of these variables. It was specifically hypothesized that fighters with higher Machiavellianism, narcissism, and psychopathy scores would have more success in winning percentages than fighters with lower Dark Triad subscale scores.

## Materials and methods

Participants

Participants for our study were recruited between January 2020 to April 2021 through email with an anonymous link for the survey sent to trainers, coaches, and fighters. Flyers were also placed in training gyms with a QR code that participants could scan to complete the survey. Our control group was recruited via email and from an undergraduate population. A total of 84 people completed the measure. Of these 84 people, 17 females were excluded due to fewer female fighters (n = 2). Two control group participants were excluded from the sample as they were considered significant outliers for college-aged non-fighters. The final sample consisted of 65 male participants. All participants provided written informed consent before participation, and the study had approval by the University’s Institutional Review Board (IRB #: 2019-566).

Our participant sample consists of individual professional fighters who are currently competing in various disciplines, including MMA (n = 32), boxing (n = 2), kickboxing (n = 1), and judo (n = 1), and male college-aged non-fighters. While the fighters' sample is composed mainly of mixed martial arts athletes, we included fighters from other related disciplines in our professional fighters group. The professional fighters group consisted of a total of 36 male fighters with ages ranging from 20 to 38 (M = 30.03, SD = 3.873) and 29 male college-aged non-fighters with ages ranging from 19 to 34 (M = 23.83, SD = 3.536). Of the fighters, 17 identified as White or Caucasian (47.2%), seven as Black or African American (19.4%), four as Asian or Asian American (11.1%), and eight as other (22.2%); 5 fighters identified as Hispanic, Latino, or Spanish Origin (13.9%). 

A majority of the comparison group identified as White or Caucasian (n = 18, 62.1%); of the remaining participants, four identified as Black or African American (13.8%), four as Asian or Asian American (13.8%), and three as other (10.3%). Nine participants in the comparison group identified as Hispanic, Latino, or Spanish Origin (31%). Of the professional MMA fighters, 18 actively competed in one of the big three promotions (UFC n = 10, Bellator n = 4, ONE Championship n = 4), while the remaining 18 competed in smaller promotions (Titan FC, LFA, etc.) (Table 1). 

**Table 1:** Demographics of Fighter and Non-Fighter Sample.

Demographics		Fighter’s		Non-Fighters	
		n	%	n	%
White or Caucasian		17	47.2	18	62.1
Black or African American		7	19.4	4	13.8
Asian or Asian American		4	11.1	4	13.8
Hispanic, Latino, or Spanish Origin		5	13.9	9	31
Other		8	22.2	3	10.3
Total		36		29	

Protocol

Participants completed the Short Dark Triad (SD3) [8]. Although research on the triad has continued to expand [9], some researchers may have been deterred by the combined length of the available measures. 

The SD3 is a self-report measure consisting of 27 items that were developed to measure qualities of Machiavellianism (“It’s not wise to tell your secrets”; “I like to use clever manipulation to get my way”), subclinical psychopathy (“I like to get revenge on authorities”; “Payback needs to be quick and nasty”), and subclinical narcissism (“People see me as a natural leader”; “Many group activities tend to be dull without me”). Participants responded to the 27 items on a 5-point scale from 1 (strongly disagree) to 5 (strongly agree). The scores of the measure divide into three subscales (narcissism, Machiavellianism, and psychopathy). Higher scores on the subscales reflect more significant levels of that respective personality trait.

Participants interested in the study were asked to follow a hyperlink in our email to complete our survey through the online software provided by Qualtrics. Participants were provided with information about the study during informed consent. The complete battery consists of four questionnaires measuring emotion self-regulation, the Dark Triad, trait anxiety, sensation seeking, and one self-efficacy question that equals 125 items total, excluding items from the demographic questionnaire. Understanding that the athlete’s and the college-aged control participant’s time is valuable and limited, we aimed to keep our battery at a reasonable number of items and estimated that it would take participants approximately 15 to 20 minutes to complete. However, participants were not restricted to time constraints to complete the measure and could do so on their own time. Upon completion, participants were thanked for their participation and debriefed. The participants did not receive any financial compensation, and participation in the study was voluntary.

Statistical analysis

All data were analyzed using IBM Corp. Released 2020. IBM SPSS Statistics for Windows, Version 27.0. Armonk, NY: IBM Corp for Mac. All p-values < .05 were considered statistically significant, and two-tailed p-values were reported. Mean scale scores were calculated by taking the mean score of the items and reverse scoring when necessary. The analysis method utilized was an independent-samples t-test comparing Machiavellianism, narcissism, and psychopathy in male professional fighters and the college-aged group of male non-fighters to test our first hypothesis. To test our second hypothesis, bivariate correlations were used to examine the relationships between variables, including Machiavellianism, narcissism, and psychopathy, and winning percentages for the fighters.

## Results

An independent-samples t-test was conducted to compare SD3 subscale scores between professional male fighters and college-aged male non-fighters. Results showed no significant differences in the SD3 subscale scores between the professional fighters and the college-aged control group of male non-fighters. Although, the SD3 subscale of psychopathy was trending towards significant, with fighters demonstrating higher scores in the psychopathy subscale (M = 22.1, SD = 66.7) compared to the comparison group of college-aged control group of non-fighters (M = 19.1, SD = 7.2), t(63) = 1.7, p < .09 (Table 2). 

**Table 2:** Results of Independent Samples T-Test Examining SD3 Subscales Between Fighters and Non-Fighters.

SD3 Subscales		Fighter’s		Non-Fighters		t (63)	p	Cohen’s d
		M	SD	M	SD			
Machiavellianism		27.53	6.74	26.86	9.04	.340	.734	.08
Narcissism		30.86	8.16	27.59	9.81	1.469	.147	.36
Psychopathy		22.06	6.67	19.07	7.23	1.729	.089	.43

These results suggest that the professional fighters and the comparison group of the college-aged control group of male nonfighters showed relatively similar scores concerning their Dark Triad personality traits. 

Our second hypothesis examined if there was a relationship between SD3 subscale scores and winning percentages (Table 3) for professional fighters. Based upon the results of the study, we found no significant correlation between Machiavellianism r(34) = -.073, p = .671; narcissism r(34) = .164, p = .338, and psychopathy r(34) = -.153, p = .373. 

**Table 3:** Descriptive Statistics and Correlations for SD3 Subscales and Fighter Winning Percentage. *p <.05

Variables	n	M	SD	1	2	3	4
Fight Winning Percentage	36	.77	.16	-			
Machiavellianism	36	27.23	7.79	-.073	-		
Narcissism	36	29.40	9.02	.164	.712*	-	
Psychopathy	36	20.72	7.03	-.153	.724*	.568*	-

These results suggest no connection between a fighter's winning percentage and their score levels on the Dark Triad personality traits.

## Discussion

Prior studies have indicated that individual athletes scored higher on the dark triad than team athletes [4,5]. Our results differ, as the individual fighters did not score higher in the dark triad compared to the general population. Research [6] highlighted how self-enhancing opportunities and glorification motivate those with higher narcissism more than those with relatively low narcissism. Given the nature of the sport, MMA provides an excellent chance for self-enhancing opportunities, we would assume higher levels of dark triad scores in the fighters, but this did not appear to be the case in the present study.

Previous research indicated that opportunities for self-enhancement and glorification encouraged individuals with high narcissism to put forth a better effort [6]. Contrary to this finding, our research demonstrated no significant correlations between winning percentages for professional fighters and Machiavellianism, psychopathy, or narcissism. It was expected that these three traits could potentially benefit fighters and be reflected in their winning percentage [6]. There did not appear to be a connection between the winning percentage and the Dark Triad.

The results of this research highlight the reality of a stigmatized group of athletes. MMA athletes have been described as "human cock fighters" and portrayed as violent and delinquent members of society [1]. The first MMA events had to occur on Indian Reserves because they were outlawed in many states. New York state had MMA outlawed up until 2016 despite the sport's popularity. Meanwhile, MMA is still illegal in some European countries, such as Norway. Although the sport is as popular as it's ever been and has entered the mainstream, there is still strong opposition to the practice and competition of MMA. This research shows that combat sports athletes are no different in some of the most undesirable personality traits to non-athletes. While their profession can be seen as brutal, violent, and vicious, the fighters themselves do not possess personality traits reserved for some of the most delinquent members of society. 

While we have considered many factors during our research, we understand that limitations and additional factors may impact a study. The first limitation that we are aware of is our small sample size. We initially planned to recruit upwards of 60 fighters. While the battery is relatively brief, with an estimated completion time of 20 minutes, the athlete may have had limited availability or desire to complete the questionnaires during recruitment. A fighter in the middle of a training camp is expected to have far less free time than a fighter who is not actively training for a fight. We did not consider where the fighter may have been in their preparation for a fight when taking the questionnaire. Prior research indicated a correlation between physiological arousal before a competition and the personality traits of agreeableness and conscientiousness [10]. This suggests that a fighter approaching a fight may have different responses than if they were not in training camp or just beginning their training camp. While we could make assumptions based on the average lengths of training camps and their fight schedule, it would have been beneficial to have the fighters explicitly state what stage they were at with their training. Future research is warranted to help examine fighter proximity to competition and score impacts. 

## Conclusions

Previous research indicated that Machiavellianism and psychopathy emerged as significant and unique correlates of symptoms of aggression. In addition, previous research showed that individual sports athletes score higher on the Dark Triad. However, our research showed that both the professional fighters and the college-aged non-fighters had no significant differences in Machiavellianism, narcissism or psychopathy scores. The discrepancy may be due to the sample size not being large enough to detect the differences between the two groups, as evidenced by the low observed power. Also, these conflicting results may be due to prior research being done on the general population and not professional athletes. According to our results, fighters are no different from the general population in factors that make up the Dark Triad. Thus, this research demonstrates that the findings from similar and previous studies are not warranted by the present study.
